# A Current Diagnostic and Therapeutic Challenge: Tinea Capitis

**DOI:** 10.3390/jcm13020376

**Published:** 2024-01-10

**Authors:** Ana Ion, Liliana Gabriela Popa, Elena Porumb-Andrese, Alexandra Maria Dorobanțu, Raluca Tătar, Călin Giurcăneanu, Olguța Anca Orzan

**Affiliations:** 1 Faculty of Medicine, ‘Carol Davila’ University of Medicine and Pharmacy, 020021 Bucharest, Romania; ralu.tatar@gmail.com (R.T.); calin.giurcaneanu@umfcd.ro (C.G.); olguta.orzan@umfcd.ro (O.A.O.); 2 Department of Dermatology, ‘Elias’ University Emergency Hospital, 011461 Bucharest, Romania; anaion00@yahoo.com (A.I.); alexandramdorobantu@gmail.com (A.M.D.); 3 Department of Dermatology, ‘Grigore T. Popa’ University of Medicine and Pharmacy, 700115 Iasi, Romania; 4 Department of Plastic Reconstructive Surgery and Burns, ‘Grigore Alexandrescu’ Clinical Emergency Hospital for Children, 011743 Bucharest, Romania

**Keywords:** tinea capitis, kerion, systemic antifungal therapy, terbinafine, griseofulvin

## Abstract

Tinea capitis is a dermatophyte scalp infection with a marked prevalence among the pediatric population. However, in the last few years, its epidemiology has changed due to increasing population migration worldwide. Host-specific and environmental factors contribute to the pathogenesis of tinea capitis. Clinically, tinea capitis may present as a subtle hair loss accompanied by scalp scaling, alopecia with scaly patches, or alopecia with black dots. A more severe form of tinea capitis is represented by kerion celsi, which clinically presents as a tender plaque covered by pustules and crusts. If left untreated, this dermatophytic infection may resolve with permanent scarring and alopecia. The pathological changes found in tinea capitis are reflected by a spectrum of clinical changes. Zoophilic infections typically prompt an extensive inflammatory reaction, while anthropophilic dermatophytoses often lack inflammation and result in more persistent lesions. Tinea capitis typically requires systemic antifungal therapy. Griseofulvin, terbinafine, itraconazole, and fluconazole are the main antifungal agents used. Currently, the duration of antifungal therapy varies based on the clinical presentation and type of dermatophyte involved. Through the reported cases and literature review, we aim to emphasize the importance of the early recognition of atypical variants of tinea capitis in immunocompetent children for the prompt initiation of systemic antifungal therapy, minimizing the need for prolonged treatment. Additionally, we emphasize the importance of regular laboratory testing during systemic antifungal therapy, particularly liver enzyme tests, to prevent adverse events, especially in cases requiring long-term treatment.

## 1. Introduction

Tinea capitis is a dermatophyte scalp infection with a marked prevalence among the pediatric population. However, its epidemiology has changed in the last few years due to increasing population migration worldwide [1]. Tinea capitis is caused by two main species of fungi: *Trichophyton* and *Microsporum* [1]. Host-specific and environmental factors contribute to the pathogenesis of tinea capitis [1]. Clinically, tinea capitis may present as a subtle hair loss accompanied by scalp scaling, alopecia with scaly patches, or alopecia with black dots [2]. A more severe form of tinea capitis is represented by kerion celsi, which clinically presents as a tender plaque covered by pustules and crusts; without a correct and prompt therapeutic approach, kerion celsi may resolve with permanent scarring and alopecia [2].

## 2. Materials and Methods

We aimed to emphasize the importance of tinea capitis, a dermatophyte infection that poses a significant diagnostic and therapeutic challenge in the pediatric population. We described two cases of tinea capitis in children. The first case required long-term systemic antifungal therapy despite a typical dermatophyte infection contracted from a cat and showed slow resolution in an otherwise healthy patient. The second case is particularly interesting because the patient had a severe clinical presentation in the form of kerion celsi, nowadays a rare variant of tinea capitis; he did not have any comorbidities and did not associate specific environmental factors such as precarious hygienic conditions and low socio-economic status. Of note, as seen in our case presentations, long-term systemic antifungal therapy may be required to achieve resolution of the infection, which further implies careful monitoring of liver function tests; moreover, cutaneous adverse reactions from systemic antifungal therapy may also be observed. We also conducted a narrative review focused on the shift in the epidemiology of tinea capitis, the sometimes atypical clinical presentations, and the resulting therapeutic approach for such cases.

## 3. Current Status of Tinea Capitis Infections

The epidemiological pattern of tinea capitis has undergone significant alterations in recent years, primarily due to migration and immigration [3,4]. This has led to changes in the prevalence, incidence, and distribution of the disease [3,4]. Recently, there has been a noticeable increase in infections caused by *Microsporum canis* (*M. canis*) in certain regions of Europe. Furthermore, *Trichophyton tonsurans* (*T. tonsurans*) has spread in urban communities in the United States of America and Western Europe, particularly in the United Kingdom and France [3,4]. Moreover, *T. tonsurans* has spread to West Africa and South America [5,6]. The rise in tinea capitis because of *T. tonsurans* has presented a challenge to community infection control in most areas [7].

The initial phase of hair shaft or epidermal invasion in tinea capitis involves a strong adhesion between keratinocytes and fungal cells, occurring immediately after the transfer of fungal cells from one host to another [8]. After the apposition phase, the dermatophytes develop modified cells which aid in invading the hair shaft [8]. In the invasion phase, the fungal cells produce a broad range of proteolytic enzymes which are active in neutral, acidic, or alkaline environments [9]. The human skin has several defense mechanisms against fungal invasion, such as the expression of human β-defensins (hBDs), cathelicidin LL-37, and dermcidin, antimicrobial peptides of the innate defense system [10]. These peptides protect against viruses, bacteria, and cutaneous infections caused by *Candida albicans* and dermatophytes [10]. Additionally, medium-chain-length fatty acids in sebum play a critical role in inhibiting the growth of dermatophytes, especially in scalp-related infections [11]. This particular mechanism is believed to be involved in the pathogenesis of tinea capitis in children and teenagers as the composition of unsaturated fatty acids that make up sebum changes during development [11]. It has been demonstrated that leukocytes, specifically human neutrophils, and macrophages play a crucial role in the body’s antifungal defense mechanism. Neutrophils can destroy up to 60% of fungi within two hours, with macrophages destroying around 20% in a similar time frame [12]. Additionally, molecular studies have identified several genes associated with susceptibility to tinea capitis. These genes play a role in macrophage regulation, keratin expression, leukocyte activation and migration, cutaneous permeability, and wound repair [13]. The clinical changes observed in cases of tinea capitis reflect the pathological changes that occur [11]. In the case of zoophilic infections, which are spread from animals to humans, patients may experience extensive inflammatory reactions [11]. On the other hand, in cases of anthropophilic dermatophytoses, which are transmitted from human to human, the lesions often lack inflammation and are more persistent [11].

Tinea capitis presents several distinct patterns that vary in etiology and clinical presentation. The ectothrix variant is caused by *Microsporum* species and affects the hair shaft at the mid-follicle level [14]. This type of infection causes hair shaft breakage 2–3 mm above the scalp, accompanied by scaly and inflamed skin [14]. In contrast, the enodothrix variant is caused by *T. soudanense*, *T. tonsurans*, *T. violaceum*, or *T. rubrum*; it is characterized by hair shaft breakage at the scalp level, with a black dot ringworm pattern and swollen hair stubs [14]. The favic variant, caused by *T. schoenleinii*, an anthropophilic dermatophyte, is characterized by air spaces within the hair shafts and the formation of large clusters of fungal hyphae at the base of the hair follicles [14].

Infections caused by *M. canis* exhibit inflamed and pruritic patches of alopecia with broken-off hairs [11]. However, infections caused by *T. tonsurans* and *T. violaceum* result in hair loss with minimal inflammatory response and a characteristic black dot formation at the surface of the scalp [11]. The most severe inflammatory reaction, kerion celsi, is usually caused by *T. verrucosum* or *T. mentagrophytes* [11]. It is characterized by the development of a tender, inflammatory mass with a purulent discharge from the follicles, often accompanied by lymphadenopathy [11]. Tinea capitis caused by *T. schoenleinii*, known as favus, is now seen sporadically; clinically, yellow crusts known as scutula are observed at the base of the hair shafts in favus cases [11]. In long-standing cases, scarring alopecia and patches of normal hair may be found in between [11].

Kerion celsi is an inflammatory form of tinea capitis, which may result from endothrix infections with *T. tonsurans* in urban areas or zoophilic ectothrix infections with *M. canis* [15]. The pronounced inflammation in kerion celsi is due to a T cell-mediated hypersensitivity reaction to the dermatophyte [16]. Clinically, kerion celsi presents as a tender, erythematous, suppurative mass with associated loco-regional lymphadenopathy and alopecia [16]. It is important to note that kerion celsi can often be misdiagnosed as a bacterial infection, leading to unnecessary systemic antibiotic therapy or surgical interventions [16]. A delayed therapeutic approach may result in permanent hair loss [16].

Tinea capitis can be diagnosed through direct microscopy and cultures [14]. Samples are obtained through scraping or using a brush or swab [14]. In recent years, dermoscopy has become a valuable diagnostic tool, enabling a closer examination of the scalp; for infections caused by *M. canis*, dermoscopy reveals elbow-shaped and dystrophic hairs, as well as broken hair at different levels, while in cases of *T. tonsurans* infections, comma-shaped hairs have been observed in the affected areas [14]. Therefore, dermoscopic examination can provide a provisional diagnosis during the initial evaluation of the patient [14].

Tinea capitis typically requires systemic antifungal therapy. Griseofulvin, terbinafine, itraconazole, and fluconazole are the primary antifungal agents used for treatment. There is a lack of established recommendations concerning the optimal duration of systemic antifungal therapy for tinea capitis. In Table 1, we present the most prevalent approach used when determining the appropriate course of treatment for tinea capitis. Topical agents often fail to penetrate the hair shaft effectively and eliminate infections [17]. Furthermore, the use of topical antifungal agents alone warrants caution since it may contribute to the development of carriers, who exhibit minimal symptoms and clinical signs but remain mycologically positive, thereby increasing the likelihood of transmitting the infection [17].

Griseofulvin was the first available oral agent for the treatment of dermatophytosis [11]. It is derived from *Penicillium* spp. and has a fungistatic effect by inhibiting various cellular processes, including nuclear acid synthesis, cell mitosis, and microtubule function [11]. It is believed to exert its fungistatic activity in several ways, including the inhibition of nuclear acid synthesis, cell mitosis, and interference with the function of microtubules [11]. In terms of pharmacokinetics, the absorption of griseofulvin may be enhanced by fatty meals, and its peak plasma levels occur four hours following oral administration; it is metabolized by the liver and excreted in urine, with a half-life of 9 to 21 h [11]. Griseofulvin can be detected in the outermost layer of the epidermis, the stratum corneum, shortly after ingestion [11]. In addition to its fungistatic activity, it has been shown to possess anti-inflammatory properties and vasodilatory effects when administered in increased doses [11]. Despite the availability of alternative treatments, many practitioners still consider griseofulvin a first-line therapy for tinea capitis [11]. Notably, the drug is well-tolerated, particularly in children, with a recommended dose of 10 mg/kg/day over a period of six to eight weeks using the microsized formulation. However, it is important to note that griseofulvin may not be available in pediatric form, such as a liquid or small tablet size, in some countries [18]. The recommended treatment duration for ectothrix infections is at least six weeks [18]. In cases where the infection is caused by *T. schoenleinii* and *T. tonsurans*, longer courses of treatment and, in some instances, even higher doses of griseofulvin (20 mg/kg/day) may be required [18]. Cutaneous eruptions, headaches, and gastrointestinal reactions are among the most common side effects of griseofulvin. Furthermore, the medication has been shown to interact with phenobarbital, oral contraceptives, and anticoagulants [18].

Terbinafine is a fungicidal drug, classified as an allylamine, that belongs to a new class of ergosterol biosynthesis inhibitors [19]. It is particularly effective against dermatophytes, both in vitro and in vivo, exhibiting potent antifungal properties [19]. The mechanism of action of allylamines consists of the inhibition of the early steps of ergosterol synthesis [19]. Regarding pharmacokinetics, terbinafine undergoes hepatic metabolism that may cause hepatotoxic effects, posing safety concerns with standard dosing across various populations [19]. A randomized controlled trial from 2011 by Deng S et al. aimed to compare the efficacy and safety of terbinafine and griseofulvin in the management of tinea capitis in the pediatric population in Western China [20]. The study found that terbinafine effectively and safely treated scalp infections caused by *T. Tonsurans*, *T. violaceum*, and *Arthroderma vanbreuseghemii* [20]. A two to four-week course of terbinafine was as effective as a four-week course of griseofulvin [20].

Itraconazole has an efficacy similar to those of griseofulvin and terbinafine against *Microsporum* and *Trichophyton* infections [15]. Itraconazole is highly keratinophilic and lipophilic, which enables it to be used as pulse therapy [21]. Usually, one to three pulses of itraconazole are required to treat tinea capitis caused by *Trichophyton* spp. However, more extensive treatment may be necessary for pediatric infections caused by *M. canis* [21,22]. The recommended dose for continuous therapy in pediatric patients ranges from 2 to 4 mg/kg/day [23]. One of the advantages of itraconazole is its availability in oral solution form, which is particularly useful for small children who may have difficulty swallowing capsules [24]. In 2021, Zhou YB et al. conducted a study designed to promote a novel itraconazole pulse therapy regimen for tinea capitis in children [24]. The authors demonstrated the efficacy and safety of administering 6–10 mg/kg/day of itraconazole with a three-week interval between pulses [24]. However, it is important to note that the study was limited to a small case series, and the authors acknowledge the need for further research involving a larger cohort of children to enable a more accurate comparison with traditional therapeutic regimens [24].

Fluconazole represents a viable alternative therapeutic option for the management of tinea capitis in children [25]. A clinical trial by Gupta AK et al. comprised 61 subjects, of whom 60 exhibited complete cure both clinically and mycologically after eight weeks of fluconazole therapy administered once weekly [25]. The adverse events were limited to mild, unspecified gastrointestinal symptoms experienced by three of the 61 children, while one patient exhibited reversible and asymptomatic altered liver functions [25].

**Table 1:** Systemic antifungal therapy for tinea capitis in children.

Antifungal Agent	Dosing Regimen
Griseofulvin	10 mg/kg/day for six to eight weeks (or 20 mg/kg/day in some *T. schoenleinii* and *T. tonsurans* infections) [1]
Terbinafine	Depending on weight: <10 kg 62.5 mg/day, 10–20 kg 125 mg/day, >20 kg 250 mg/day- four weeks [26]
Itraconazole	2–4 mg/kg/day for four to six weeks [23]
Fluconazole	3–6 mg/kg/day for six to eight weeks [27]

## 4. Case Reports

### 4.1. Case Report 1

**Figure 1:**
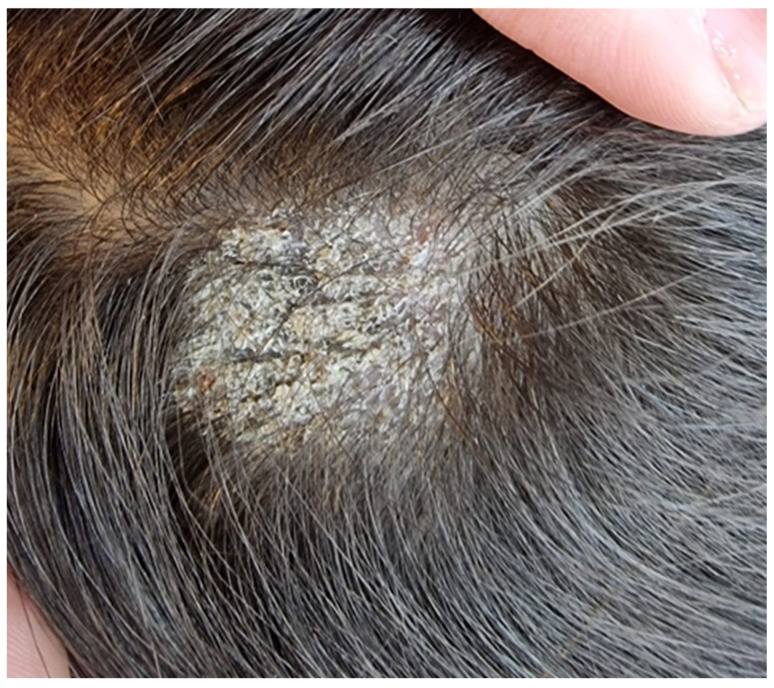
Scaly, alopecic patch on the scalp.

**Figure 2:**
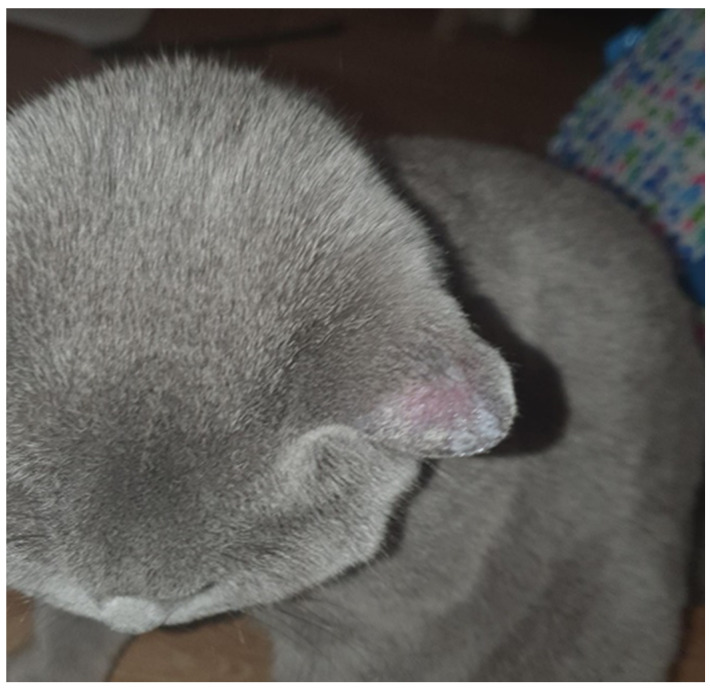
Alopecic plaque on the left ear of the patient’s cat.

**Figure 3:**
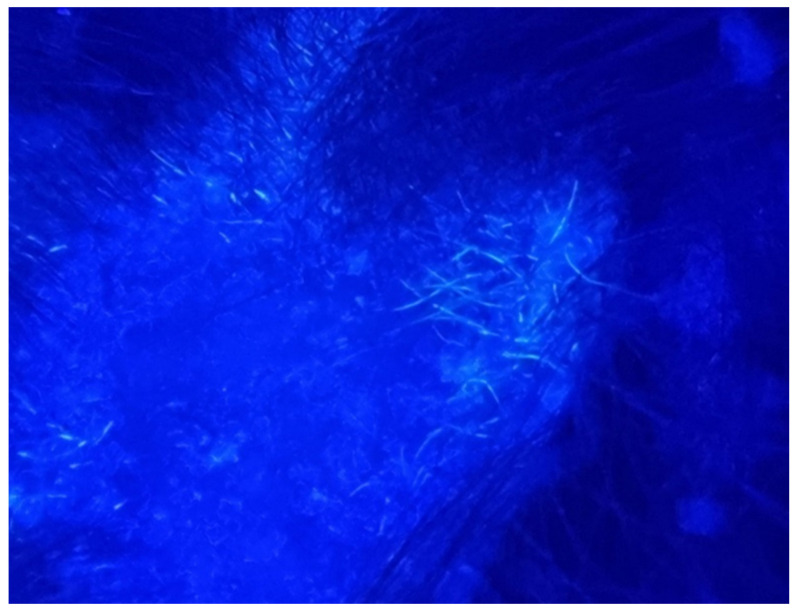
Wood’s lamp examination. Blue-green characteristic fluorescence was observed.

**Figure 4:**
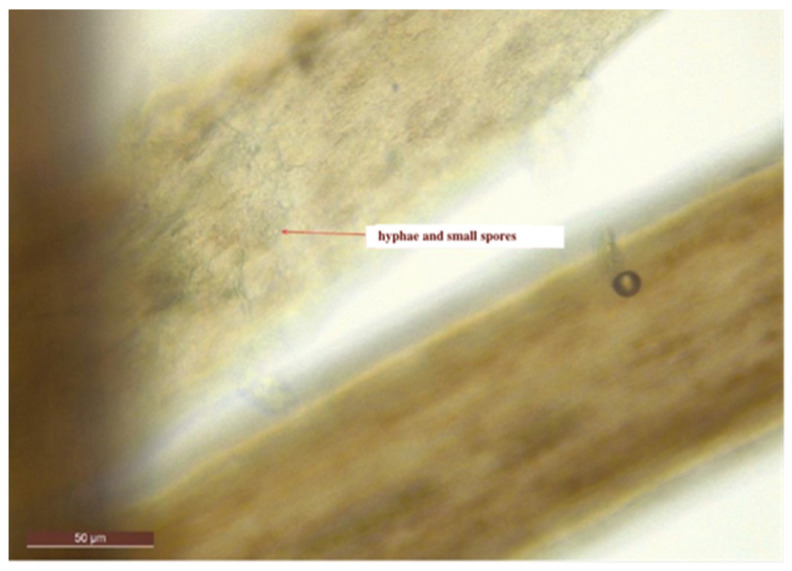
Direct mycologic examination of the hairs treated with 20% potassium hydroxide solution showing hyphae and small spores.

**Figure 5:**
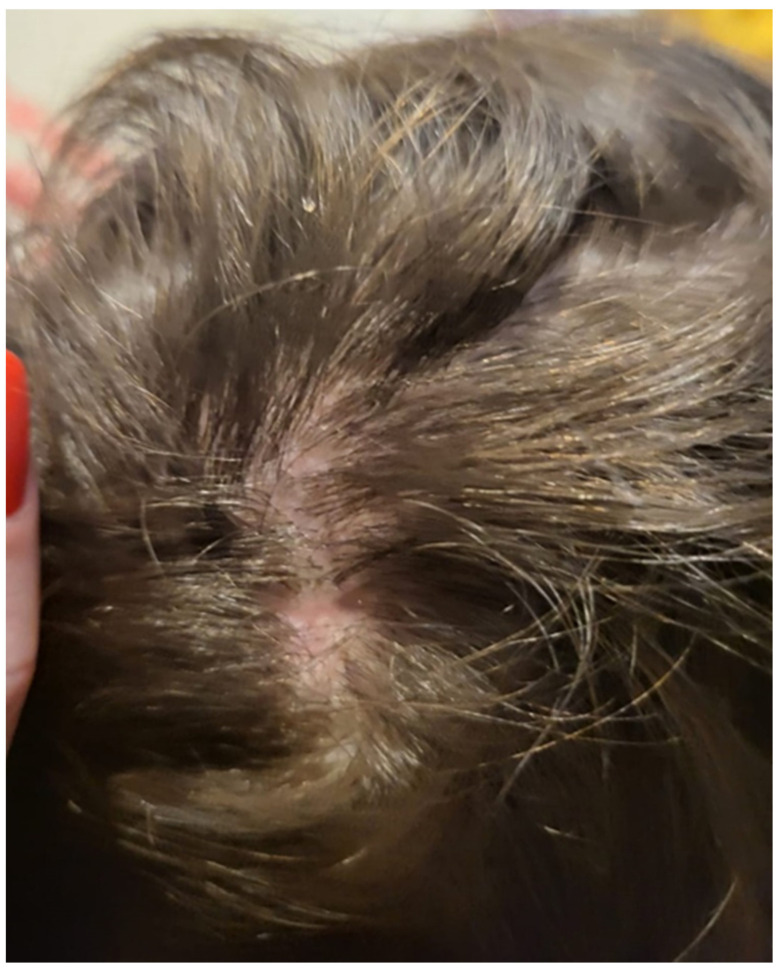
Significant clinical improvement after six months of systemic antifungal therapy with griseofulvin.

We report the case of a seven-year-old female patient who presented with a pruritic and scaly alopecic plaque that had developed over the course of three weeks (Figure 1). Regarding the patient’s history, the girl’s mother mentioned the presence of a similar plaque on their cat’s left ear (Figure 2). Upon examination of the lesion using a Wood’s lamp, we observed a blue-green fluorescence, suggestive of *Microsporum canis* infection (Figure 3). Direct mycological examination under the optical microscope, following the application of a 20% KOH solution, detected the presence of hyphae and small spores within the hairs (Figure 4). After correlating the patient’s medical history, clinical examination, and mycologic results, we made the diagnosis of tinea capitis caused by *M. canis*. The therapeutic approach consisted of oral administration of fluconazole syrup at a dose of six milliliters three times a day for three weeks, with subsequent clinical reevaluation. Upon follow-up, due to the absence of clinical improvement under fluconazole therapy, it was decided to switch to griseofulvin. Liver function tests were performed regularly during systemic antifungal therapy with griseofulvin; no liver dysfunction was detected. Significant clinical improvement was observed after six months of therapy with a daily dose of 125 mg of griseofulvin (Figure 5). Furthermore, mycological tests were returned negative for hyphae and small spores.

### 4.2. Case Report 2

**Figure 6:**
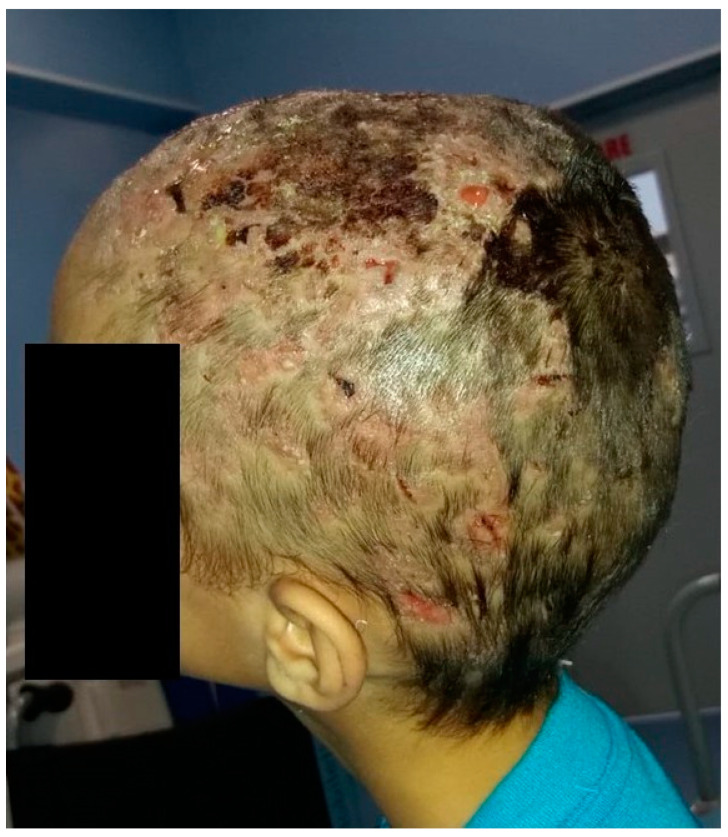
Multiple fluctuant nodules on the scalp with purulent discharge and yellow crusts associated with alopecia, consistent with the kerion celsi variant of tinea capitis.

**Figure 7:**
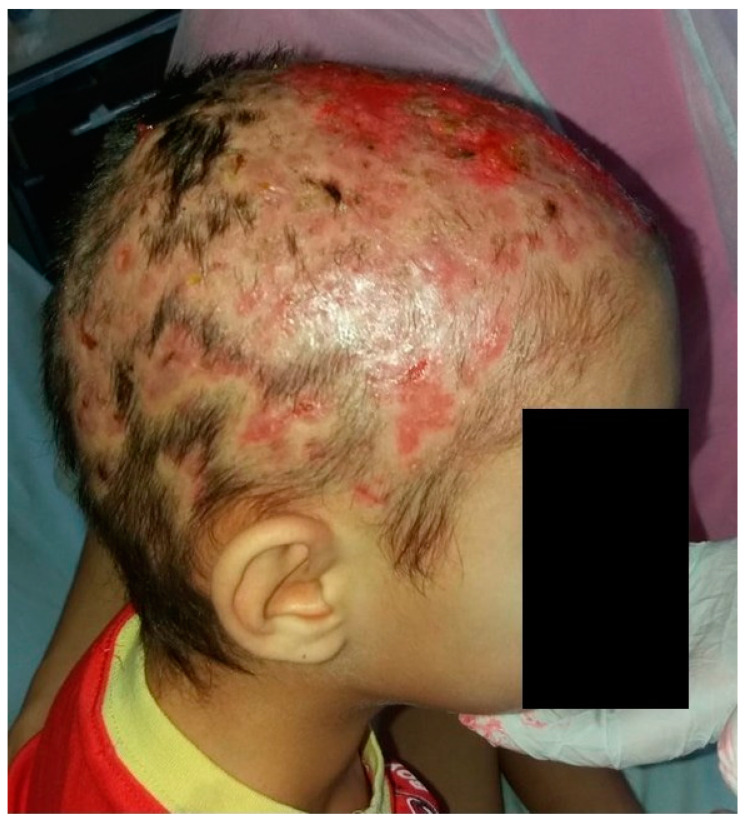
Clinical aspect of the scalp after surgical debridement of the necrotic tissue.

**Figure 8:**
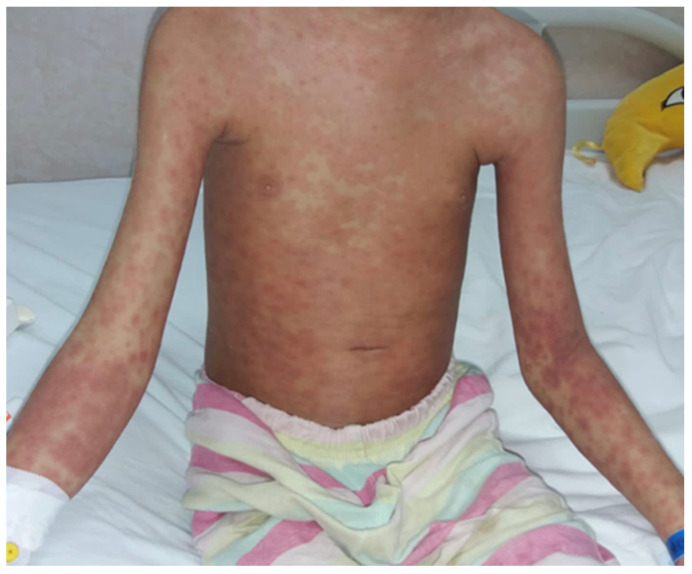
Polymorphic erythematous eruption on the trunk and upper extremities.

We report the case of an eight-year-old male patient who presented with multiple fluctuant nodules on the scalp with purulent discharge and yellow crusts associated with alopecia and regional lymphadenopathy. The clinical lesions appeared approximately one month before presentation. Regarding the patient’s history, his brother had similar clinical lesions on the scalp. Previously to the present admission, the patient received systemic antibiotic therapy with meropenem intravenously for seven days, which led to the aggravation of the disease, with extensive scalp involvement. The patient was otherwise healthy, with regular laboratory tests and no comorbidities. Following clinical evaluation and trichoscopy, the diagnosis of superinfected tinea capitis, specifically the kerion celsi variant, was established (Figure 6). The therapeutic approach consisted of surgical debridement of the necrotic tissue followed by systemic therapy comprising intravenous fluconazole at a dosage of 6 mg/kg per day, terbinafine 125 mg once a day for seven days, gentamycin at a dosage of 7.5 mg/kg per day (2.5 mg/kg administered every eight hours), and meropenem 10 mg/kg per day every eight hours for ten days (Figure 7). Subsequent to the patient’s discharge, oral fluconazole was prescribed at a dosage of 400 mg/day for four weeks. Following the initiation of therapy, the patient developed a polymorphic erythematous eruption characterized by the presence of polycyclic, urticarial, and targetoid-like macules on the trunk and upper extremities (Figure 8). The lesions resolved shortly upon administration of systemic corticosteroids. It is plausible that the adverse reaction may have been triggered by the administration of terbinafine, given that the lesions emerged soon after the medication was administered. Furthermore, the patient’s medical history indicates no prior adverse reactions to fluconazole, gentamycin, or meropenem. Liver function tests were performed upon admission, followed by subsequent tests on the third day of systemic antifungal therapy and at the end of the treatment period. The results of the examinations were indicative of normal liver function. Although clinical remission was attained, there was noticeable scarring on the scalp.

## 5. Discussion

Early recognition of the distinct clinical aspects of tinea capitis in children, particularly amidst the significant epidemiological changes, is crucial to avoid any delay in the initiation of antifungal systemic therapy. Long-term antifungal therapy may be deemed necessary for severe cases, as evidenced in Case Report 1. Laboratory tests should be performed before, during, and after antifungal therapy, with liver function tests being of particular importance. Exsudative variants of tinea capitis may affect immunocompetent patients, and cutaneous adverse reactions may occur during systemic antifungal therapy, as shown in Case Report 2. We further discuss the epidemiologic shift of dermatophytoses in recent years, several notable atypical clinical presentations of tinea capitis reported in the literature, and the safety and efficacy of long-term systemic antifungal therapy in children.

The epidemiology of dermatophytoses is influenced by many factors and has changed throughout the years [28]. Taxonomically, the dermatophytes belong to the *Onycogenales* order and *Arthrodermataceae* family and were, traditionally, divided into three genera, namely, *Trichophyton*, *Microsporum*, and *Epidermophyton*. Recently, both the taxonomy and nomenclature of the dermatophytes was thoroughly revised [29]. The well-known division intro three genera has been expanded to nine genera (*Microsporum*, *Tricophyton*, *Epidemophyton*, *Nannizzia*, *Lophophyton*, *Arthroderma*, *Ctenomyces*, *Guarromyces*, and *Paraphyton*) [29]. Various publications have reported on the changes in the incidence and distribution of dermatophyte species in different regions. For instance, in the Middle East, the *T. rubrum* complex has been found to be the leading cause of skin and nail infections, accounting for over 90% of cases [30]. A study on onychomycosis from 2020 from North West Greece showed that the *T. rubrum* complex was the most frequently isolated dermatophyte (74.4%), followed by *T. mentagrophytes interdigitale* (21.4%) [31]. Migration plays a key part in the demographic changes [28]. For instance, *T. tonsurans*, which originates from Southeast Asia and Australia, has spread to Latin America [28]. It subsequently proliferated through immigrant workers in North America and spread to other regions worldwide, including Africa [28]. Wilmington M et al. described the increase in tinea capitis infections caused by *T. tonsurans* in the San Francisco area between 1974 and 1994, showing a dramatic increase: while in 1970, 41.7% of cases were caused by *T. tonsurans*, in 1990, the prevalence increased to 87.5% [32]. According to a recent report by Grigoryan KV et al. in 2019, *T. tonsurans* was responsible for 95% of tinea capitis cases in children [33]. The report also revealed that two other dermatophytes, *T. violaceum* and *T. soudanense*, were also found to cause tinea capitis in children, especially in African immigrants [33]. A study conducted by Mashiah J et al. in 2016 reported an outbreak of tinea capitis among children of African immigrants in Israel. The study performed from 2010 to 2014 included 145 children and concluded that the primary causative agents were *M. audouinii* and *T. violaceum* [34]. The correlation between the prevalence of dermatophytoses and climate factors is particularly interesting [35]. It is widely accepted that due to the proliferation of dermatophytes in warm, humid conditions, the occurrence of dermatophytoses is likely to be more prevalent in tropical and subtropical regions [35].

*T. indotineae* is a recently identified dermatophyte species responsible for refractory dermatophytoses in various parts of the world, particularly in the Indian subcontinent, affecting patients of all ages and sexes [36]. The emergence of *T. indotineae* has raised significant concerns due to its association with extensive dermatophytosis and resistance to terbinafine and other antifungal agents, leading to numerous treatment failures [36,37,38,39]. The reports suggest that *T. indotineae* may have zoonotic origins, and its emergence is likely due to the widespread misuse of antifungal agents [40,41]. Furthermore, *T. indotineae* has been found to exhibit resistance to azole compounds, such as itraconazole and voriconazole, further complicating treatment options [41]. *T. indotineae* causes a widespread cutaneous eruption characterized by inflammation, itching, and dermatophytosis affecting the face, trunk, and inguinal area [37].

Identification of *T. indotineae* has posed a significant challenge, necessitating the development of rapid and cost-effective methods for its detection [42,43]. Such techniques include real-time polymerase chain reaction (PCR) and MALDI-TOF mass spectrometry, which works by generating protein fingerprint signatures from whole bacterial cells, allowing for the identification of bacteria and fungi at the species level within minutes [44,45]. This technique has shown promise as an alternative to conventional phenotypic identification methods for most bacterial and fungal pathogens commonly found in clinical samples [12,46]. In addition to microbial identification, MALDI-TOF MS has been used to study antibiotic resistance mechanisms [47].

Correspondingly, research has spotlighted the genetic mutations responsible for terbinafine resistance in *T. indotineae*, particularly in the squalene epoxidase gene, which is a target for antifungal agents [48]. The genetic considerations and clinical presentations of antifungal resistance in dermatophytes, including *T. indotineae*, have been thoroughly investigated, underscoring the need for alternative therapies and improved antifungal susceptibility testing [49]. Currently, itraconazole is the preferred drug to treat dermatophytosis caused by *T. indotineae* [50]. The recommended dosage is 100 mg of itraconazole twice a day for 4 to 8 weeks, and in some cases, it may extend up to 12 weeks [51].

*T. indotineae* is a significant emerging pathogen causing refractory dermatophytoses, particularly in the Indian subcontinent, and its resistance to multiple antifungal agents poses a challenge for clinical management. Efforts to understand its genetic resistance mechanisms and develop rapid detection methods are crucial for effectively controlling and managing infections.

*T. benhamiae*, a dermatophyte belonging to the *Arthroderma benhamiae* species, was first documented in the Far East, specifically in Japan, but the incidence has increased worldwide in the last 15 years [52,53]. *T. benhamiae* has emerged as an increasingly prevalent zoophilic dermatophyte, with a strong incidence in Germany, where it is the most commonly observed pathogen implicated in zoophilic dermatophytosis, particularly in pediatric cases [54,55]. It has been identified as an important zoonotic pathogen, especially in children, and has been associated with highly inflammatory tinea in individuals who have had previous contact with guinea pigs, rodents, and other pets [56,57]. *T. benhamiae* causes various dermatophytoses, including tinea capitis, tinea corporis, tinea manuum, and tinea faciei, often resulting in severe inflammation, particularly in children and immunocompromised patients [58]. Moreover, it has been associated with severe inflammatory responses. During *A. benhamiae* infections, the secretion of various cytokines from keratinocytes has been observed, suggesting its potential to trigger significant immunological reactions in the host, being associated with secondary bacterial infections and complications such as kerion celsi [54,59,60].

*T. benhamiae* strains have been classified as yellow and white based on their morphology. The yellow strains have a pleated mycelium and a yellow-orange reverse and grow slowly [53,61]. They produce few microconidia and no macroconidia or spiral hyphae on Sabouraud agar [53,61]. This makes it difficult to differentiate them from *Microsporum canis*, which is also often yellow in appearance but has different microscopic characteristics.

The strains displaying the white phenotype are either powdery or floccose in appearance and exhibit a yellow, orange, or brown reverse. These strains are characterized by a rapid growth rate and the presence of multiple spherical to clavate microconidia. Their macroconidia are sparse, smooth, thin-walled, and clavate to cigar-shaped, and spiral hyphae may occasionally be present [53]. The white phenotype of *T. benhamiae* must be differentiated from *T. mentagrophytes*, which has more spherical microconidia and frequent spiral hyphae [53].

Additionally, molecular identification studies have classified *T. benhamiae* within the *T. benhamiae* series and other related species, such as *T. verrucosum*, *T. concentricum*, and *T. erinacei* [62]. This classification has been supported by genetic diversity analyses, which have revealed the intraspecies variability and mating behavior of *A. benhamiae*, indicating its close relationship with other species within the *T. benhamiae* series [60]. Moreover, *T. benhamiae* has been the subject of extensive research to understand its prevalence, propagation factors, and transmission patterns. The current treatment of *T. benhamiae* dermatophytoses entails the administration of systemic antifungal agents, notably terbinafine, fluconazole, and itraconazole. Scarring is a common complication [55].

The emergence of *T. benhamiae* as a significant cause of dermatophytosis, especially among children, underscores the need for additional research to understand its epidemiology, dynamics of transmission, and pathogenic mechanisms.

Tinea capitis may display atypical clinical presentations that sometimes delay the proper therapeutic approach, which may lead to a more extended period of antifungal therapy. Anane et al. documented a case of an 11-year-old girl with diffuse scalp scaling and thick, scaly patches but no alopecia who had a history of tinea amiantacea treated with a keratolytic shampoo [63]. Scales and altered hairs were collected for laboratory testing [63]. Direct microscopic examination in 20% potassium hydroxide revealed an endothrix invasion with septate hyaline hyphae [63]. The culture on Sabouraud glucose agar stored at 27 °C yielded colonies of white appearance with ramifications that submerged into the agar after twenty days [63]. Microscopic examination of these colonies showed multiple branched hyphae, termed favic chandeliers, terminal hyphal dilation that gave rise to nail head shape structures (favic nails), and chlamydoconidia [63]. Based on the clinical and mycological data, the diagnosis of tinea favosa caused by *T. schoenleinii* was confirmed [63]. The patient underwent systemic antifungal therapy with griseofulvin (20 mg/kg/d) and ketoconazole in the form of foaming gel once a day for a period of twelve weeks [63]. Despite being compliant with the treatment, the patient experienced grey hair and permanent alopecia patches [63]. On follow-up, the mycological examination was negative twice, at two months (day 60) and three months (day 90) after the treatment was completed [63]. Unfortunately, the source of the contamination could not be identified through the epidemiologic survey [63].

In 2017, Grijsen ML and de Vries HJC reported a case of an eight-year-old male patient who presented with a tender, erythematous mass on his scalp [16]. The mass had purulent discharge and measured approximately 5 × 6 cm [16]. The boy also had alopecia, ipsilateral lymphadenopathy, and small black dots [16]. The patient had initially experienced a round patch of hair loss for several weeks, which was treated with topical ketoconazole 2%. However, the lesion progressed despite therapy. Systemic antibiotic therapy was administered, but the patient exhibited no clinical improvement [16]. He also had a few round, erythematous and scaly patches on his left cheek, trunk, and extremities [16]. His father displayed similar lesions on his arm, and their feline pet had developed bald patches. Fungal hyphae were observed after the microscopic examination of skin scrapings. A diagnosis of kerion celsi was suspected, and the patient was initiated on griseofulvin at a dose of 20 mg/kg daily for twelve weeks, with a favorable response [16]. After four weeks of the initial presentation, *T. tonsurans* was isolated from the culture, confirming the diagnosis [16].

In 2023, Ikutama R et al. reported the case of a 13-year-old male who was a judo club member at his high school [64]. The patient presented with multiple erythematous papules on the occipital area a week prior to his visit. These papules subsequently developed into a 10 × 10 cm swelling, accompanied by purulent discharge and pain [64]. The lesions developed into a 10 × 10 cm swelling showing purulent discharge pain [64]. He was diagnosed with kerion celsi caused by *T. tonsurans* according to the fungal examination [64]. Therapy with terbinafine was initiated at a dose of 125 mg daily for six weeks [64]. At the end of the treatment, a brush test was performed, and it came back negative. Out of the 18 judo club members, four tested positive on the brush test, while the patient’s parents tested negative [64]. The authors emphasized the need for testing and treatment of family members and colleagues, when necessary, to prevent the spread of infection [64].

Chokoeva AA et al. reported the case of a five-year-old male patient who presented with a severe cutaneous and scalp desquamation, along with an indurated ringworm plaque in the temporal area [65]. Following a diagnostic biopsy, the patient’s histology report indicated a psoriasiform pattern, although no Munro abscesses or Kogoj pustules were detected [65]. Subsequent mycological laboratory tests revealed infection with *T. verrucosum* [65]. The patient was treated with a 10% salicylic acid topical formulation and received systemic treatment with terbinafine at a dose of 125 mg daily [65]. Significant clinical improvement was observed within the following two months [65]. The authors of this case report emphasize the crucial role played by the host’s immunological response in the pathogenesis of clinically atypical manifestations caused by zoophilic dermatophytes, specifically *T. verrucosum* [65]. Additionally, they highlight the potential occurrence of an “Id reaction”, which can result in diagnostic errors and delayed therapy. Therefore, it is essential to be aware of this reaction to minimize diagnostic errors and ensure timely treatment [65].

Terbinafine may induce cutaneous adverse effects that can manifest in various forms, including pruritus, urticaria, erythematous eruptions, acute generalized exanthematous pustulosis, subacute cutaneous lupus erythematosus, papulosquamous conditions such as lichenoid-drug eruptions or pityriasis rosea-like lesions, and in rare cases, Stevens–Johnson syndrome [66,67,68]. Other systemic adverse effects of terbinafine include gastrointestinal symptoms such as nausea, vomiting, and epigastric pain, as well as headaches. Laboratory tests may show neutropenia and hepatic dysfunction [69]. Patients should be closely monitored during therapy with terbinafine to identify any change in their liver function tests [69]. Rare but severe adverse reactions have been reported with systemic terbinafine use [70].

In 2010, Bansgaard N et al. conducted a study in Denmark on 263 patients (140 women and 123 men) who had reported adverse reactions to systemic terbinafine. The study aimed to analyze these reactions over a 10-year period [70]. Of the 263 subjects, 78 of them experienced severe adverse effects. Notably, 32% (25/78) of the subjects experienced the effects in the first five years, while 68% (53/78) experienced them in the last five years of the study [70]. Several patients presented with more than one adverse reaction, resulting in 493 total reactions, of which 197 were classified as severe adverse reactions (SAEs) [70]. The adverse events were further classified into system-organ classes, with cutaneous adverse events being the most prominent class, with 60 patients (30% of patients) who experienced erythema multiforme, exfoliative dermatitis, Steven–Johnson’s syndrome, and toxic epidermal necrolysis (TEN) [70]. Of all the reports, 15% (29 patients) exhibited hepatobiliary disorders, along with elevated liver enzymes [70]. Other disorders, such as metabolic, cardiac, respiratory, hematologic, infectious, psychiatric, and vascular disorders, were also identified, although each had fewer than 10 cases [70]. Notably, one case of death was reported during the study period, involving an 86-year-old male patient who passed away due to pancytopenia while undergoing treatment for a fungal skin infection [70]. The authors emphasize the importance of considering the impact of fungal disease on the quality of life of patients, the potential benefits of systemic antifungal therapy, and the availability of alternative therapeutic options [70]. Moreover, the study authors insisted on the importance of following prescription guidelines and administering systemic antifungal therapy solely to microbiologically confirmed mycoses. [70]. Patients must also be informed of potential adverse effects associated with therapy [70].

In a study by Sabzghabaee AM, the safety and efficacy of terbinafine were evaluated in a small cohort of pediatric patients suffering from tinea capitis in Iran [19]. The study included 30 patients who were administered systemic terbinafine based on their body weight [19]. Patients with a body weight less than 20 kg were given 62.5 mg terbinafine, while patients with a body weight between 20 and 40 kg were given 125 mg for eight weeks [19]. Of the 30 patients, 30% reported gastrointestinal symptoms (nine cases), 16.7% reported pruritus (five cases), 6.7% reported skin rash (two cases), and 6.7% reported headache (two cases) [19]. None of the subjects showed any signs of hepatic dysfunction [19]. The authors concluded that terbinafine was effective in treating tinea capitis in pediatric patients without any severe side effects [19].

## 6. Conclusions

Tinea capitis still represents a current diagnostic and therapeutic challenge in pediatric patients. The clinical presentation of tinea capitis can vary, and in some cases, it may mimic other conditions, such as alopecia areata or dissecting cellulitis, or present with abscess formation, making accurate diagnosis challenging. Tinea capitis, particularly kerion celsi, is often misdiagnosed as a bacterial infection. Additionally, the etiological agents of tinea capitis can vary by region, and the emergence of atypical pathogens in certain populations further complicates its management. The variations in the distribution of tinea capitis across different areas underscore the necessity for area-specific approaches in its diagnosis and control. Our study presents two cases of severe tinea capitis in children from good socio-economic backgrounds. Notably, our findings show no association between the prevalence of tinea capitis and factors such as low socio-economic status and high population densities. Thus, these results prompt a reevaluation of current approaches to the diagnosis and control of tinea capitis, highlighting the importance of a targeted approach tailored to the specific needs of individual regions.

The administration of systemic antifungal therapy is the preferred therapeutic approach, given its established efficacy. However, careful monitoring of liver enzymes and other laboratory tests is imperative to prevent adverse events, particularly in cases where prolonged systemic antifungal therapy is necessary. Such diligent monitoring ensures that any potential complications arising from the therapy can be promptly detected and managed, thereby improving patient outcomes.

## Data Availability

Data are contained within the article.
